# Interleukin-33: Its Emerging Role in Allergic Diseases

**DOI:** 10.3390/molecules23071665

**Published:** 2018-07-09

**Authors:** Wen Ding, Gui-Lin Zou, Wei Zhang, Xing-Ning Lai, Hou-Wen Chen, Li-Xia Xiong

**Affiliations:** 1 Department of Pathophysiology, Medical College, Nanchang University, 461 Bayi Road, Nanchang 330006, China; lmwl5@live.com (W.D.); z1293612123@gmail.com (G.-L.Z.); laixingning99@outlook.com (X.-N.L.); chenhouwen@sina.com (H.-W.C.); 2 Gannan Medical University, Rongjiang New Area, Ganzhou 341000, China; zhangweign@outlook.com

**Keywords:** interleukin-33, allergic diseases, target therapy, asthma, Th2-type immunity

## Abstract

Allergic diseases, which include asthma, allergic rhinitis (AR), chronic rhinosinusitis (CRS), atopic dermatitis (AD), food allergy (FA), allergic keratoconjunctivitis, seriously affect the quality of life of people all over the world. Recently, interleukin-33 (IL-33) has been found to play an important role in these refractory disorders, mainly by inducing T helper (Th) 2 immune responses. This article reviews the mobilization and biological function of IL-33 in allergic disorders, providing novel insights for addressing these hypersensitive conditions.

## 1. Introduction

Interleukin-33 (IL-33), which belongs to the larger family of damage-associated molecular pattern molecules, has been considered as an ‘alarmin’. It is released to alert the immune system by first-line cells, such as tissue epithelial cells, following exposure to exogenous stimuli, including allergens [1,2]. This cytokine is also expressed in endothelial cells, fibroblasts, smooth muscle cells, osteoblast, adipocytes and several immune cells including macrophages and dendritic cells (DCs) [1,3,4,5]. IL-33 was firstly identified as a nuclear factor of high endothelial venules in 2003 [6] and later was identified as a member of the IL-1 family [3,7]. Thus, IL-33 is not only an intracellular nuclear factor but also an extracellular cytokine. Intracellularly, IL-33 is found to possess transcriptional repressor properties and nuclear IL-33 reduces nuclear factor kappaB (NF-κB)-triggered gene expression to dampen pro-inflammatory signaling by sequestering nuclear NF-κB [8]. In contrast, IL-33 up-regulates the p65 subunit of the NF-κB complex in the nucleus and promotes inflammation in endothelial cells [9]. Extracellularly, IL-33 acts as an inflammatory cytokine and exerts its biological effects via binding to a heterodimer receptor complex consisting of suppression of tumorigenicity 2 (ST2) and the IL-1 receptor accessory protein (IL-1RAcP).

Allergic diseases are regarded as common, refractory diseases around the world. More importantly, they are more common in urbanized societies as they are associated with modern lifestyle [10]. The pathogenic immunity of allergic diseases is characterized by refractory inflammation and Th2-mediated immune responses. Recently, IL-33 has been considered as an emerging key factor in the development of allergic diseases. Increasing genomewide association studies have reported the loci of IL-33 gene plays important roles in susceptibility of several diseases such as asthma and allergic rhinitis. It can aggravate allergic diseases by inducing pro-inflammatory cytokines production and Th2 type immune cell activation. This review summarizes current findings regarding IL-33 and discusses its pathogenic role in allergic diseases including asthma, allergic rhinitis (AR), chronic rhinosinusitis (CRS), atopic dermatitis (AD), food allergy (FA), allergic keratoconjunctivitis. We also discuss the therapeutic potential of targeting IL-33 and ST2.

## 2. IL-33 Signaling Pathway

**Figure 1:**
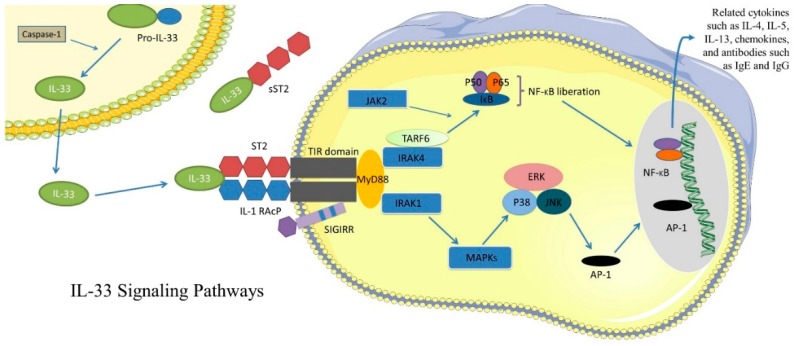
IL-33 signaling pathway. IL-33 binds with ST2 transmembrane isoform and IL-1 receptor accessory protein (IL-1RAcP), leading to the downstream activation of several signaling pathways. MyD88 recruitment at TIR domain is essential for IL-33-ST2-mediated signaling cascade. On one hand, the downstream activation of TRAF6 and IRAK4 proteins activates the inhibitor of nuclear factor-κB kinase (IKK) complex, leading to NF-κB activation. On the other hand, in the absence of TRAF6, downstream activation of MAPKs leading to activation of AP-1. Next, NF-κB and AP-1 bind to DNA and induce the expression of various Th2-related cytokines, chemokines and antibodies. sST2 acts as a decoy receptor and results in the competitive inhibition of IL-33 biological activity. Single Ig IL-1R-related molecule (SIGIRR) acts as negative regulator for TIR signaling, leading to the suppression of IL-33/ST2 signaling pathways.

IL-33 receptor ST2 is a member of toll like receptor/Interleukin-1 receptor (TIR) superfamily and highly expressed in immune cells including macrophages, eosinophils, DCs, mast cells (MCs), basophils, NK cells, innate lymphoid group 2 cells (ILC2s), Th2 lymphocytes and B cells [3,11,12], as well as the endothelium, epithelium cells and fibroblasts [7,13]. Mechanically, IL-33 binds with ST2 and IL1RAcP to form a heterodimer receptor complex and then activates the Myeloid differentiation factor 88 (MyD88) adapter protein at TIR domain. The downstream molecular activities include the recruitment and activation of IL-1R-associated kinase 1 (IRAK1), IRAK4 and tumor necrosis factor (TNF) R-associated factor 6 (TRAF6) [7,10]. These signaling molecules may in turn lead to the activation of different signaling pathway including NF-κB, p38 mitogen-associated protein kinase (MAPK)-extracellular signal-regulated kinase (ERK)/c-Jun N-terminal kinase (JNK)-activator protein-1 (AP-1), thereby inducing the production of inflammatory cytokines [12,14] (Figure 1). In addition to the membrane form of ST2, soluble ST2 (sST2) is produced by alternative splicing and acts as a decoy receptor to suppress IL-33-induced immune responses.

## 3. The Role of IL-33 in Allergic Diseases

### 3.1. The Role of IL-33 in Asthma

Asthma is a chronic inflammatory, allergic lung and bronchus disease, which generally marked by airway hyperresponsiveness (AHR), tissue remodeling and excessive Th2 inflammation. It is characterized by infiltration of eosinophils, elevated Th2 cytokine levels, and IgE. The house dust mite (HDM, mainly *Dermatophagoides pteronyssinus*) is the main allergen which is involved in several allergic diseases, such as asthma and AR. Interestingly, a recent study in children with severe asthma has reported that the lower airways of patients displays a dominant Th1 signature [15]. This indicates that the immune environment of asthma may be not fully understood. Notably, IL-33 expression is up-regulated in the bronchial mucosa of asthmatic patients related to disease severity [16,17]. Its receptor ST2 expression is increased in endobronchial specimens and blood cells of severe asthmatic and associated with multiple indicators of Th2 inflammation [18,19]. Moreover, IL-33 polymorphisms influence the susceptibility to asthma. A study aiming to identify the association of IL-33 polymorphisms with wheezing phenotypes of asthma identified that rs4742170 and rs7037276 are associated with intermediate-onset wheeze, and rs1342326 is associated with persistent wheeze [20]. In a European birth cohort study, Schröder et al. [21] have found that rs928413 and rs1342326 are relevant for asthma and hay fever development in German children. Moreover, combined heterozygotes and minor allele homozygotes of these two polymorphisms are associated with decreased regulatory T cells (Tregs) and increased suppressor of cytokine signaling-3 [21]. Interestingly, a rare variant in IL-33, rs146597587-C allele, is associated with lower eosinophil tallies, and reduced risk of asthma and allergic rhinitis in Europeans. It disrupts a canonical splice acceptor site before the last coding exon and leads to IL-33 loss-of-function [22]. These studies indicate IL-33 may play an important role in asthma process.

**Figure 2:**
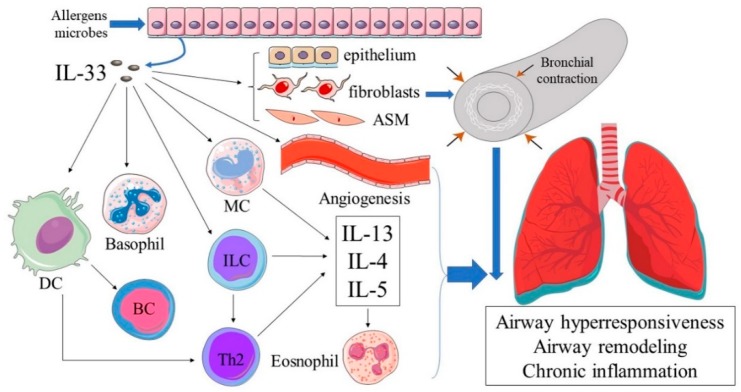
IL-33 in asthma pathology. IL-33 is involved in the initiation and perpetuation of airway hyperresponsiveness (AHR), airway remodeling and inflammation in asthma process.

More specifically, increased expression of IL-33 are associated with increased reticular basement membrane thickness in endobronchial specimens from children with severe therapy-resistant asthma [23]. Angiogenesis is another feature of airways remodeling in asthma. In patients with allergic asthma, IL-33 modulates migration of hematopoietic progenitor cells as allergic inflammatory responses [24]. Further study in mice model has observed that serial intranasal challenge with IL-33 induces airways angiogenesis by promoting expression of multiple angiogenic factors [25]. In vitro, IL-33 promotes the airway recruitment and function of circulating fibrocytes [16]. Further studies have found IL-33 promoted collagen synthesis from asthmatic fibroblasts both in vitro and in vivo [23,26]. In addition, high-level IL-33 expressed in bronchial epithelium and airway smooth muscle (ASM) is correlated with AHR [27]. After stimulation with IL-33, bronchial epithelium significantly expressed ST2 on its surface and released IL-17F dependent on ST2-ERK1/2 signaling pathway [28]. In mice model, IL-33 induced ASM contraction and AHR via upregulation of MC-derived IL-13 [27]. These results may explain that IL-33 levels correlate inversely with the lung function (forced expiratory volume in the first second) of asthmatics [17]. Accordingly, IL-33 favors permanent structural changes and AHR by activating several signaling pathways and inflammatory cells (Figure 2).

IL-33 integrates both innate and adaptive immunity in asthmatic immune activation. Although Th2 cells-mediated adaptive immunity is important in asthma process, innate immune mechanisms involving IL-33 are also important in asthma [29]. Kim et al. found that IL-33 induced IL-5 and IL-13 production by natural helper cells and NKT cells to promote AHR development [29]. In addition, ILC2s are described as a novel member of the ILC family. This cells group are lineage-negative cells that induce innate type 2 responses by producing the Th2-type cytokines in response to IL-33 and IL-25. IL-33 and ILC2s levels are upregulated in the airways of asthmatic patients and correlated with disease severity [30]. In mice model, knock out ILC2s or blockade of IL-33-ST2 signaling have resulted in reduction of airway inflammation and AHR [30]. In premature infant mice following neonatal hyperoxia, IL-33 signaling and ILC2s are also vital for the induction of asthma-like features [31]. Moreover, interconnected circuits between ILC2s and epithelial cells are required for asthma chronicity. Epithelial IL-33 induces ILCs to release IL-13, which upregulates IL-33 receptor and helps IL-33 autoinduction, thus establishing a feed-forward circuit [30].

IL-33 mediates Th2-type responses through ST2 expression on the surface of several immune cells. In vitro and in vivo, IL-33 induces DCs recruitment and activates DCs to produce IL-6, TNF, CCL17 and to promote lymphocytes to produce the Th2 cytokines [32]. Moreover, IL-33 enhances the ligation between allergen-specific IgG and FcγRIII on DCs to maintain development of Th2-mediated responses [33]. MCs are also vital in asthmatic development. IL-33 activates MCs to produce Th2-type cytokines, such as IL-13, via MyD88-integrated crosstalk amongst the IL-33 receptor ST2, TLR4 and FcεRI [34]. Interestingly, IL-33-ST2 signaling induces Th17 response via MC-dependent Th17-differentiation [35]. In addition, IL-33 exacerbates allergic airway bronchoconstriction by increasing synthesis, storage, and secretion of serotonin from the MCs [36]. However, IL-33/ST2-dependent MCs response may act as a protective role in the development of AHR. Zoltowska et al. found that MC-deficient mice engrafted with ST2-deficient bone marrow-derived mast cells (BMMCs), following exposition to HDM, shown an exacerbated development of AHR and reduced prostaglandin E2 (PGE2) levels in BALF [37]. The authors explained that IL-33/ST2-dependent MCs induction of PGE2 might had protective effect on AHR. In addition, Morita et al. reported that lung inflammation was exacerbated and Treg cells count was decreased in MC-deficient mice [38]. Further study in vitro found IL-33-stimulated MCs expressed IL-2 and the latter promoted expansion of numbers of Treg cells, thereby suppressing development of papain- or IL-33-induced airway eosinophilia [38].

IL-33-ST2 axis is involved in the activation and function of other immune cells, which is the potent mechanisms underlying IL-33-induced asthmatic development. It’s reported that IL-33-ST2-p38 signaling is required to the production of IL-5, IL-13 by pathogenic memory Th2 cells and eosinophilic inflammation [39]. IL-33-ST2-JNK/NF-κB signaling has been shown to induce neutrophil polarization and production of IL-4, IL-5, IL-9 and IL-13 by neutrophil [40]. IL-33 also induces B cells activation and promotes the induction of total IgE and HDM IgG1 production [41]. In vitro, Treg cells lose their ability to suppress effector T cells in the presence of IL-33 [42]. Moreover, even established immunologic tolerance in the lungs is impaired after IL-33 administration in mice. In addition, Gordon et al. have found basophils are also the target of IL-33 bioactivity in asthma. In response to IL-33 stimulation, basophils express ST2 and produce Th2 cytokines such as IL-4, IL-5, and IL-13 [43].

Infections with rhinoviruses or fungal are the main basis of asthma exacerbations. Jackson et al. have reported IL-33 release and IL-33-responsive immune cells are pivotal mediators between viral infections and exacerbation of asthma [44]. IL-33 may act as a suppressor of innate antiviral immunity by down-regulating interferon (IFN)-α and IFN-λ production [45]. In addition, IL-33-mediated Th2 polarization and AHR may be the mechanisms underlying pediatric severe asthma with fungal sensitization [46]. Interestingly, IL-33-dependent ILC2 expansion, eosinophilia, Th2 cytokines expression and AHR are also important in aspirin-exacerbated respiratory disease, a severe asthma subtype [47].

Clinically, helminth parasites strongly inhibit Th2 allergy. In asthma mice model with administration of *Alternaria* extract, the soluble excretory/secretory products of *H. polygyrus* suppresses early IL-33 release, Th2 cytokines production by ILCs, as well as eosinophilia [48]. In asthma mice model with exposure to pneumonia virus, anti-IL-33 decreases Th2 inflammation by suppressing rhinovirus replication and increasing IFN-λ levels [49]. Ad5-gsgAM, an adenovirus vector carrying two mycobacterial antigens Ag85A and Mtb32, suppresses Th2 response of asthma by inhibiting IL-33/ST2 axis and favoring Treg expansion in mice [50]. In addition, blocking IL-33 activity with sST2 has significant suppressive effects on *Staphylococcus aureus*-derived serine protease-like protein D-induced allergic Th2 responses [51]. In summary, down-regulation of IL-33 production or modulating the IL-33-ST2 pathway may provide therapeutic effects in asthma, especially those severe asthma subtypes.

### 3.2. The Role of IL-33 in Allergic Rhinitis (AR) and Chronic Rhinosinusitis (CRS)

AR is an IgE-mediated type 1 allergic disease in nasal mucosa caused by nasal allergen exposure. Patients with AR or CRS often have characteristic symptoms including sneezing, rhinorrhea, nasal congestion, facial pain, and hyposmia. Furthermore, CRS can be divided into two main subtypes, CRS with nasal polyps (CRSwNP) and CRS without nasal polyps (CRSsNP), characterized by the presence or absence of polyps. AR and CRS have been considered as Th2-dominated allergic diseases and often accompany tissue eosinophilia. Perhaps because both AR and CRS are nasal allergic diseases, there is an estimated prevalence of AR of 60% in CRS patients [52]. In intermittent AR patients who are sensitive to tree and/or grass pollen, serum IL-33 is up-regulated and correlates with disease severity [53]. Interestingly, study including 24 Japanese cedar (JC) pollinosis patients and 14 HDM-sensitized patients with AR has found that IL-33 protein is not detected in the serum, but IL-33 level is increased in sinus mucosa and significantly correlated with the total nasal symptom score [54]. Moreover, test in the allele frequency and genotype distribution of each polymorphism between different subjects has found a significant association between rs1929992 T allele and JC pollinosis [55]. In nasal polyps from CRSwNP patients, Kim et al. have reported IL-33 level is positively correlated with the number of neutrophils and the expression of several Th1 and Th17 inflammatory markers [56].

IL-33 receptor ST2 is also highly expressed in the nasal mucosa of patients with AR or CRS [57,58,59], suggesting that IL-33/ST2 may play an important role in the pathogenesis of allergic nasal diseases. Moreover, many ST2^+^ eosinophils are observed in the mucosa of eosinophilic CRS (ECRS) but not in the mucosa of non-ECRS patients [60]. This conforms to findings that the mRNA and protein levels of IL-33 and ST2 are significantly higher in nasal polyps of CRSwNP and ECRS [58]. Moreover, ST2 level is positively correlated with symptom and CT scores in eosinophilic CRSwNP and with Th2 cytokine expression in sinonasal mucosa [61]. These studies indicate that IL-33 and its receptor ST2 may play important roles in the pathogenesis of CRSwNP and ECRS. However, recent study has shown that tissue IL-33 level is lower in the CRSwNP group compared with the control groups, and reported a significant negative correlation between IL-33 levels and clinical CT scores [62], although serum IL-33 levels are increased. In addition, sST2 concentration is significantly increased in the nasal lavages collected from AR patients during the pollen allergy season. Moreover, nasal sST2 level is negatively correlated with nasal symptoms [63], suggesting a potential regulatory role of sST2 in AR inflammation.

AR is the immediate-type allergy caused by IgE-MCs-mediated early-phase responses (5–30 min after allergen exposure). In addition, Th2 cytokines and chemokines mediate the migration of immune cells into nasal mucosa, typically eosinophilia, which cause the late-phase responses of AR (6–24 h after allergen exposure) resulting in tissue damage and remodeling. In vitro, IL-33 expression is up-regulated in nasal epithelial cells (NECs) after treatment with the bacteria-associated molecule CpG [64], IFN-γ, the toll-like receptor 9 ligand ODN2006 [57,59]. In vitro, IL-33 induces IL-8 and GM-CSF expression in human NECs [57]. In response to allergens, IL-33 is released from NECs and induces the release of IgE-mediated histamine and chemo-attractants from FcεRI1 MCs and basophils, respectively [52,65]. Interestingly, AR patients sensitized to HDM or mugwort allergen have distinct phenotypic and functional profiles in ILC2s frequencies [66]. ILC2s induce Th2 responses and tissue remodeling by producing Th2 cytokines in response to IL-33/ST2 signaling [52,58]. IL-33-ST2-ILC2s axis is also important in allergic fungal rhinosinusitis, which is a subtype of CRS caused by bacterial or fungal infection. In addition, IL-33 can induce Th2 cytokine production in Th2 cells, MCs, basophils and eosinophils, suggesting that IL-33 has the potential to induce Th2-type allergic inflammation [59,65,67]. Moreover, IL-33-ST2-induced Th2 response also interacts with Th17 immune response [68,69], showing multiple T cells responses in the progression of allergic nasal diseases.

After ragweed exposure, mice shows aggravated AR symptoms, nasal Th2 activation, increased level of serum ragweed-specific IgE and the infiltration of eosinophils and basophils in the nasal mucosa, which aren’t showed in IL-33- and ST2-deficient mice [65,70]. IL-33 and its receptor are the novel therapeutic targets for AR and CRS treatments. Serum levels of IL-33 are significantly decreased after standardized specific immunotherapy treatment [71]. Moreover, 2-aminoethoxy-diphenyl borate reduces IL-33-induced hypersecretion of Muc5b from glandular cells by controlling intracellular Ca^2+^ concentration [72]. Anti-IL-33 treatment significantly reduces the nose-scratching events and ameliorated skin denudation with decreased eosinophilic infiltration and decreased levels of serum total and OVA-specific IgE [73]. Histopathological changes are also significantly improved by anti-IL-33 treatment [56]. In addition, intranasal administration with anti-TNF-α IgY results in decreased production of IL-33 and OVA-specific IgE levels in the peripheral blood and nasal lavage fluid [74].

### 3.3. The Role of IL-33 in Atopic Dermatitis (AD)

AD is a common chronic inflammatory skin disease characterized by Th2 immune signatures. Patients often suffer from severe pruritus, relapsing eczematous lesions. In skin, several mediators released by immune cells regulate the complex network of AD. Moreover, recent evidences focus on an important role of IL-33/ST2 pathway in skin. IL-33 and ST2 levels are elevated in epidermis of AD patients [75] and high-level IL-33 in serum significantly correlates with excoriation and xerosis scores [76]. IL-33 is found at an increased level in basal and thickened lower spinous layers of the epidermis from patients with Netherton syndrome, which is characterized by allergic responses in skin and diffuse desquamation [77]. Moreover, suprabasal keratinocytes and endothelial cells were the main sources of IL-33^+^ and ST2^+^ cells [78,79]. In vitro, IL-33 induces keratinocytes to produce the cytokine IL-6 and the chemokines CXCL-8/IL-8, CCL-20, CCL-17, CCL-5, and CCL-2 dependent on ERK, p38 MAPK, JNK, and NF-κB signaling pathways [75]. Similarly, IL-33-ST2 signaling enhances IL-6 and AD-related chemokines secretion from eosinophils and fibroblasts [80]. Moreover, IL-33 down-regulates the β-defensin 2 expression in human primary keratinocytes [81] and influences the susceptibility to bacterial superinfection in acute allergic eczema of AD.

In vitro, IL-33-induced DC promotes the differentiation from naïve Th cells into Th2 cells and maintain memory Th2 cells, leading to the increased production of IL-5, IL-9, and IL-13 [82]. Further study in mice model has shown IL-33 mediates vitamin D3 analog MC903-induced AD via ST2-MyD88 signaling in DCs [83]. In addition, allergen exposure and filaggrin deficiency amplify the expression of ST2 and IL-33 in BMMC [78]. Interestingly, the mechanisms by which AD occurs are complex. Both Th1 and Th2 immune responses are involved in chronic inflammation of AD. In vitro, TNF-α and IFN-γ induce expression of IL-33 mRNA and protein in human dermal fibroblasts, HaCaT keratinocytes, macrophages, and HUVEC endothelial cells [78,79,84]. Meanwhile, CD4^+^ T cells respond to IL-33 and induce the release of IFN-γ, therefore driving skin inflammation towards chronic responses [79]. Moreover, blockade of IL-33 attenuates ear swelling in allergic contact dermatitis (ACD), a murine model of AD, but not the sensitization with oxa, suggesting initially upregulated IL-33 is secreted as alarmin without functioning in the sensitization stage, then IL-33 sustains chronic inflammation for the up-regulated TNF-α and IFN-γ [84]. Even in specific pathogen-free conditions, transgenic mice with IL-33 gene suffers from itchy dermatitis and develops phenotypes that closely resemble the features of AD as IL-33 expressed in skin induces infiltration of eosinophils, MCs and ILC2s and IL-5 production by ILC2s [85]. In addition, IL-33 instructs vaccinia virus dissemination in skin and internal organs of Eczema Vaccinatum, which is a life-threatening complication of exposure to smallpox vaccination in AD patients [86].

Skin-derived ILC2s expressing ST2 are enriched in lesional skin biopsies from AD patients [87]. In addition to mediate immune response, IL-33 and ILC2s play important roles in the skin barrier dysfunction. Most patients with AD display a reduced expression of filaggrin, involucrin and loricrin, which are indispensable elements of the epidermal barrier [87]. In the early stage of allergy and tissue injury, up-regulated IL-33 in response to stimulator is necessary for the reduction of tissue integrity damage via ST2 expressed in ILC2s, suggesting IL-33 and ILC2s may be critical for maintaining epithelial integrity and tissue homeostasis [88]. However, Seltmann et al. have reported that IL-33 down-regulates filaggrin expression in keratinocytes and impairs the skin barrier [89]. Moreover, IL-33-induced null mutation of filaggrin gene lead to the down-regulation of E-cadherin, which down-regulates the expression of the ILC2 signature cytokines IL-13 and IL-5, and reduces ILC2 proliferation [87]. In vitro models, IL-33 stimulation shows a significant concentration-dependent downregulation of involucrin and filaggrin, with reduced total epidermal thickness and the thickness of nucleated epidermal layers (total epidermal thickness without the stratum corneum [90]). In conclusion, IL-33 may down-regulate transcription of genes from members of the epidermal differentiation complex, ultimately leading to impaired epidermal growth and maturation.

These studies reveal IL-33 contributes to AD or ACD and offer a possibility to predict the therapeutic benefits of IL-33 in skin allergic diseases. Several reports have suggested IL-33- or ST2-deficient mice shows diminished AD syndrome with decreased immune cells infiltration and Th2 cytokine expression. Anti-mouse IL-33 antibody treatment results in the improvement of AD symptoms, as well as the reduction of cells infiltration and IgE levels [84,91]. Chrysin is proved to be a candidate which inhibited TNF-α/IFN-γ-stimulated IL-33 expression in mouse primary keratinocytes from the same model [92]. Moreover, Qingre-Qushi Recipe has significant effects in down-regulation of IL-33-ST2 signaling axis and Th2 cytokines [93]. Topical tacrolimus treatment decreases the expression of IL-33 and ST2 in mouse AD skin [78]. Although IL-33 is implicated in pro-allergic and pro-inflammatory process in AD, it may have adverse effect in other aspects. In vitro, IL-33 induces IL-2 release in murine BMMCs via MAPK activation and IL-2 significantly contributed to expansion of Tregs. The positive correlation between IL-33 and IL-2-expressing MCs is also found in oxazolone-challenged ears of mice model and skin samples obtained from patients [94].

### 3.4. The Role of IL-33 in Food Allergy (FA)

FA is an imbalanced immune disease driven by Th2 immune responses that may lead to different manifestations in skin or intestine. Importantly, it is perplexing why only some food allergic individuals who have high levels of IgE acquire susceptibility to developing life-threatening anaphylaxis. IL-33 and other Th2 cytokines levels are increased in mice exposed to intragastric peanut allergen [95]. Moreover, further study has indicated IL-33 induces DC OX40L and expand ILCs, suggesting that IL-33 signaling may play a significant role in the allergen sensitization of gut. Interestingly, more occurrence in infancy in the absence of oral exposure, indicated that alternative route may also contributes to food allergy [96]. Several reports aim to test the hypothesis that skin allergy mediated by food components promote gut sensitization. In mice, epicutaneous peanut exposure induces cytokine expression dependent on the IL-33-ST2 signaling in DCs and T cells, and resulted in the skin sensitization to the food allergens, suggesting IL-33 may mediate food allergy through skin route in early life [96,97]. In summary, IL-33 contributes to food anaphylaxis via various routes of exposure including gastrointestinal tract and skin.

After the sensitization stage, IL-33 induces gut anaphylaxis via IgE cross linking and MCs degranulation. Moreover, IL-33- or ST2-deficient mice and mice treated with sST2 were protected from anaphylaxis [98,99]. In vitro, IL-33 activates mucosal MCs to secrete IL-9, which plays a important role in anaphylaxis as it enhances intestinal mastocytosis [100,101]. In addition to MCs, IL-33 may activate ILCs and suppress Treg function that favor FA immunity [102]. Further studies have found barrier defects play key roles in IL-33-dependent anaphylaxis. Mechanical skin injury [103] or impairment of intestinal barrier integrity by the mycotoxin deoxynivalenol [104] lead to IL-33 release, the latter promotes oral anaphylaxis after epicutaneous sensitization. From these studies, it can be concluded that IL-33 may have a significant role in affiliation between the barrier defects and FA. Recently, Khodoun et al. have reported injection of an mAb to IL-33 receptor strongly inhibits FA development [105]. Accordingly, IL-33 and its receptor ST2 may be used as novel therapeutic targets to suppress MCs-associated pathogenesis in FA.

Eosinophilic esophagitis (EoE) is a chronic inflammatory disease, the pathogenesis of which is associated with allergy to food and airborne antigens. Analysis of esophageal biopsies from EoE patients has showed increased Th2 cytokines IL-5 and IL-13, CCL26, and IL-33 mRNA expression, but increased IL-33 wasn’t found in the serum [106]. In a mice model of EoE, exogenous IL-33 induces marked structural changes in the mucosa, transmural inflammation and hyperproliferation in the mouse esophagus, accompanied by eosinophil migration and activation in the early stage of EoE [106]. Interestingly, IL-33 significantly inhibits Treg cell differentiation and the regulatory factors TGF-β and IL-10, suggesting IL-33 induces loss of antigenic tolerance in EoE development [106]. In addition, it’s reported that IL-33 supports eosinophil homeostasis by inducing production of IL-5, which is crucial for supporting mature eosinophils [107]. When developed clinically, IL-33 inhibitors may be important candidates to bring therapeutic function in EoE patients.

### 3.5. The Role of IL-33 in Allergic Keratoconjunctivitis

Th2 responses in ocular surface mediate inflammation against various allergens and cause tissue damage and ocular surface infection, which lead to occurrence and exacerbation of atopic keratoconjunctivitis. It’s reported that IL-33 protein is highly expressed in human vascular endothelial cells and conjunctival epithelium in the giant papillae obtained from patients with atopic keratoconjunctivitis [108]. In vitro, IL-33 contributes to ST2 expression, IL-13 mRNA induction and p38MAPK phosphorylation in human MCs [108], production of pro-allergic cytokines TSLP and chemokines in human corneal epithelial cells [109]. These pro-allergic factors were inhibited by sST2 protein [108,109]. These findings reveal that an essential role of IL-33-ST2-Th2 signaling in the regulation of Th2-mediated allergic inflammation.

IL-33-ST2-Th2 signaling is also found activated in experimental allergic conjunctivitis models. After ragweed pollen administration, IL-33 is released and lead to the infiltration of eosinophils and CD4^+^ T cells with activated ST2 on the surface in conjunctival stroma [110]. These activations are obviously reduced in IL-33-knockout mice [111]. Furthermore, pollen-induced IL-33 production is decreased in TLR4-deficient mice and is blocked by TLR4 antibody in vitro [112]. This study has reported that pollen-TLR4 innate immune pathway in mucosal epithelium may instruct the production of pro-allergic IL-33 that in turn activates IL-33-ST2-Th2 signaling pathways as adaptive immune responses. In conclusion, IL-33 may serve as a bridge linking innate to adaptive immune responses and maintain the chronic immune response of allergic keratoconjunctivitis. IL-33 and ST2 may become novel molecular targets for the interference of allergic diseases in ocular surface.

## 4. Concluding Remarks and Perspectives

The data available in several studies suggests that IL-33 acts as a possible pathogenic role in allergic diseases (Table 1). IL-33 plays important roles in allergic diseases mainly by instructing the activation of various ST2-expressing cells and the production of several immune factors. The mechanisms underlying IL-33-mediated inflammation have been immunologically analyzed. However, much remain to be illuminated regarding the accurate functions and underlying mechanisms of the IL-33-ST2 signaling pathways in these allergic diseases. It has been suggested that IL-33–blocking drugs may be promising therapeutic agents, especially for the severe subtypes of allergic diseases, such as the infection with virus or fungus. Although several reports have shown that some natural products or compounds have suppressive function for IL-33-induced inflammation at the screening level, more studies for elucidating their specific IL-33-inhibiting potential should be conducted.

## Figures and Tables

**Table 1:** IL-33/ST2 expression and function in allergic diseases.

Diseases	IL-33/ST2 Expression (Citation)	IL-33 Function (Citation)
Asthma	IL-33 level is increased in bronchial mucosa and related to disease severity [16,17]; ST2 level is increased in blood cells [19]; ST2 level is increased in bronchial mucosa and associated with Th2 inflammation [18]; IL-33 level is decreased after treatment [48,50]	IL-33 polymorphisms influence asthma susceptibility [20,21,22]; IL-33 favors AHR and airways remodeling by activating tissue cells [23,24,25,26,27,28]; Harmful via innate and adaptive Th2 immunity mainly; Possibly protective via PGE2 and Treg activation [37,38]; Pivotal mediator in asthma exacerbation by infection with Rhinoviruses or fungal [44,45,46]
Allergic Rhinitis and Chronic Rhinosinusitis	IL-33 and ST2 levels are increased in serum and sinus mucosa and related to disease severity [53,54,55,56,57,58,59,60,61,62]; nasal sST2 level is increased and negatively correlated with nasal symptom [63]; IL-33 level is decreased after treatment [71,74]	IL-33 induces tissue eosinophilia in CRS [60,61]; Harmful in AR as Th2-type allergic inflammation [52,58,59,65,66,67,68,69]
Atopic Dermatitis	IL-33 and ST2 levels are increased in skin [75,77,78,79]; Serum IL-33 level correlates with disease severity [76]; IL-33 level is decreased after treatment [78,92,93]	Detrimental roles dependent on Th2 immunity mainly [75,80,81,82,83]; IL-33 collaborates with TNF-α and IFN-γ to maintain AD chronic inflammation [78,79,84]; IL-33 and ILC2s play important roles in skin barrier dysfunction [87,89,90]
Food Allergy	IL-33 level is increased in gut and esophageal biopsies [95,96,106]	IL-33 contributes to food anaphylaxis via various routes [95,96,97]; IL-33 plays a significant role in affiliation between the barrier defects and MC-associated FA [98,99,100,101,102,103,104]; IL-33 instructs Th2 immunity and mastocytosis in EoE [106,107]
Allergic Keratoconjunctivitis	IL-33 level is increased in conjunctival stroma [108,110]	IL-33 induces eosinophils infiltration and Th2 cytokines production [108,109,110,111,112]

## Abbreviations

The following abbreviations are used in this manuscript:

IL-33	Interleukin-33
AR	allergic rhinitis
CRS	chronic rhinosinusitis
AD	atopic dermatitis
FA	food allergy
Th	T helper
DCs	dendritic cells
ILC2s	innate lymphoid group 2 cells
NF-κB	nuclear factor kappaB
ST2	suppression of tumorigenicity 2
IL-1RAcP	the IL-1 receptor accessory protein
MCs	mast cells
MyD88	Myeloid differentiation factor 88
IRAK	IL-1R-associated kinase
TRAF	tumor necrosis factor (TNF)R-associated factor
MAPK	p38 mitogen-associated protein kinase
ERK	extracellular signal-regulated kinase
JNK	c-Jun N-terminal kinase
AP-1	activator protein-1
IKK	the inhibitor of nuclear factor-κB kinase
SIGIRR	Single Ig IL-1R-related molecule
AHR	airway hyperresponsiveness
Tregs	regulatory T cells
HDM	House Dust Mite
ASM	airway smooth muscle
BMMCs	bone marrow-derived mast cells
PGE2	prostaglandin E2
IFN	Interferon
CRSwNP	chronic rhinosinusitis with nasal polyps
CRSsNP	chronic rhinosinusitis without nasal polyps
JC	Japanese cedar
ECRS	eosinophilic chronic rhinosinusitis
NECs	nasal epithelial cells
ACD	allergic contact dermatitis
EoE	Eosinophilic esophagitis
